# Lighting Up Ca^2+^ Dynamics in Animal Models

**DOI:** 10.3390/cells10082133

**Published:** 2021-08-19

**Authors:** Nelly Redolfi, Paloma García-Casas, Chiara Fornetto, Sonia Sonda, Paola Pizzo, Diana Pendin

**Affiliations:** 1 Department of Biomedical Sciences, University of Padua, 35131 Padua, Italy; nelly.redolfi@unipd.it (N.R.); paloma.garciacasas@unipd.it (P.G.-C.); chiara.fornetto@unipd.it (C.F.); sonia.sonda@studenti.unipd.it (S.S.); paola.pizzo@unipd.it (P.P.); 2 Neuroscience Institute, National Research Council (CNR), 35131 Padua, Italy

**Keywords:** calcium imaging, calcium indicators, GECI, animal models, nervous system

## Abstract

Calcium (Ca^2+^) signaling coordinates are crucial processes in brain physiology. Particularly, fundamental aspects of neuronal function such as synaptic transmission and neuronal plasticity are regulated by Ca^2+^, and neuronal survival itself relies on Ca^2+^-dependent cascades. Indeed, impaired Ca^2+^ homeostasis has been reported in aging as well as in the onset and progression of neurodegeneration. Understanding the physiology of brain function and the key processes leading to its derangement is a core challenge for neuroscience. In this context, Ca^2+^ imaging represents a powerful tool, effectively fostered by the continuous amelioration of Ca^2+^ sensors in parallel with the improvement of imaging instrumentation. In this review, we explore the potentiality of the most used animal models employed for Ca^2+^ imaging, highlighting their application in brain research to explore the pathogenesis of neurodegenerative diseases.

## 1. Introduction

Ca^2+^ signaling pathways are involved in virtually every aspect of cellular physiology, and their dysregulation have often been documented in pathological situations. Intracellular Ca^2+^ concentration ([Ca^2+^]_i_) must be tightly controlled in terms of both space and time to generate regulated signals. This dynamic control is obtained through the orchestrated interplay of Ca^2+^ transport systems, localized in the plasma membrane (PM) and the membranes of the intracellular Ca^2+^ stores and the Ca^2+^ buffering systems.

Ca^2+^ generates versatile signals in the nervous system [1]. In neurons, fast Ca^2+^ influx occurs via voltage-gated Ca^2+^ channels upon membrane depolarization, while slower Ca^2+^ signals come from intracellular stores, which work together sin generating the dynamic regulation of brain activity. Here, Ca^2+^ signals operate over a wide temporal range to control a plethora of phenomena [2], spreading from neurotransmission [3] to plasticity [4] and gene transcription [5]. 

Besides regulating fundamental neuronal functions, Ca^2+^ signals also sustain processes relevant to the occurrence and progression of neurodegeneration [6,7,8,9]. Common, early Ca^2+^ alterations have been described across different neurological conditions, setting the basis for the hypothesis of a shared upstream Ca^2+^ dysregulation in their pathogenesis. Nevertheless, in different neurodegenerative diseases, e.g., Alzheimer’s, Parkinson’s, and Huntington’s disease, specific defective Ca^2+^ signals have been reported, originating from distinct neuronal compartments, organelles, or subdomains. These distinctive features are supposed to initiate or sustain disease pathogenesis, often in selected neuronal subtypes (e.g., dopaminergic neurons in Parkinson’s disease [7,10] and striatal neurons in Huntington’s disease [11]) or specific brain regions (e.g., the hippocampus in Alzheimer’s disease, AD [12]), that are likely more vulnerable to the defect. Thus, investigating Ca^2+^ signaling regulation and dysregulation represents a key to obtaining a comprehensive picture of disease pathogenesis, with the final aim of developing effective and specific therapeutic strategies [13]. Many of these Ca^2+^-mediated pathological features have been successfully explored in animal models. Indeed, relevant genes and fundamental biological pathways are mostly conserved through evolution [14,15,16]. Notably, most of the cellular processes involved in the pathogenesis of human disorders are conserved in animal models [17], making the latter valuable tools to investigate the mechanisms that take part in aging and neurodegeneration. 

In the 1980s, the development of the first Ca^2+^ indicators enabled the possibility to measure Ca^2+^ dynamics in living cells, opening the route for deciphering fundamental intracellular signaling pathways [18,19]. Later, genetically encoded Ca^2+^ indicators (GECIs) [20] also allowed the implementation of imaging techniques in vivo in multi-cellular animals. This represented a breakthrough for brain research since GECIs enabled the indirect measure of neuronal activity by recording the fast and transient variations in [Ca^2+^]_i_. The investigation of Ca^2+^ signals through fluorescence imaging is now of utmost importance in order to gain information regarding not only single-cell, activity-dependent [Ca^2+^]_i_ variations, but to also map neuronal population activity and functional connectivity [21,22] as well as localized Ca^2+^ dynamics from specific subcellular regions, e.g., dendritic spines [23].

In this review, we will analyze the applications of Ca^2+^ imaging in some of the most used animal models, highlighting their role in unraveling important aspects of the pathogenesis of neurodegenerative diseases.

## 2. Fluorescent Indicators for Ca^2+^ Imaging in Animal Models

Over 30 years ago, the design of the first organic fluorescent Ca^2+^ indicators represented a revolution in the field of Ca^2+^ investigations, initiating the fruitful season of cellular Ca^2+^ imaging [19]. A variety of probes have been developed since then that now cover a wide range of Ca^2+^ affinities and kinetic properties [24]. 

There are two major families of indicators that have been developed, i.e., chemical probes and GECIs. Chemical indicators (e.g., fura-2, indo-1, fluo-4), small fluorescent molecules based on BAPTA (1,2-bis(o-aminophenoxy)ethane-*N*,*N*,*N*′,*N*′-tetraacetic acid) chelating moiety, are widely used in cultured and isolated cells [25,26,27] but have been poorly exploited in live animal models [28,29,30,31], which is mainly due to tissue permeation issues. On the contrary, GECIs have been widely exploited in live animals due to the techniques available to express the engineered Ca^2+^ sensing proteins that they encode in vivo [32,33,34]. Notably, GECIs allow the monitoring of Ca^2+^ not only in the cytosol, but also in specific organelles, thanks to the addition of specific targeting moieties to their sequences [35,36,37].

GECIs include single fluorescent protein-based indicators (e.g., GCaMP), bioluminescent probes (e.g., aequorin) and fluorescence (or Förster) resonance energy transfer (FRET)-based indicators (e.g., cameleons) [38]. Among single fluorescent protein indicators, the most widely used is the GCaMP family of probes [32,33,34,39,40]. They consist of a circularly permuted variant of Green Fluorescent Protein (GFP), whose N-terminus is connected to the M13 fragment of the myosin light chain kinase. On the other side of the protein, the C-terminus ends with the Ca^2+^-binding region of calmodulin (CaM). In the presence of Ca^2+^, M13 wraps around Ca^2+^-bound CaM, and this leads to a conformational change that increases the fluorescence intensity of the molecule [41]. In recent years, efforts have been devoted to ameliorating the brightness, dynamic range, and Ca^2+^ affinity of the first indicators. As a result, a palette of GCaMP indicator variants have been developed, with a range of Ca^2+^ affinity that allows the measure of [Ca^2+^]_i_ not only in the cytosol, but also in the subcellular compartments [24]. Importantly, other fluorescent protein variants (e.g., XCaMPs) have been developed, making the GCaMP family a useful tool to measure Ca^2+^ dynamics simultaneously in different neuronal types or subcellular regions [42] (Table 1). 

Although of undiscussed value, single protein-based GECIs suffer from some sensitivity to focal plane shifts, which are caused by the movement of samples, photobleaching, and other artifacts that could lead to a change in fluorescence that is not linked to a change in [Ca^2+^]_i_. To overcome this limitation, fluorescence emitted upon excitation at the isosbestic point of the GCaMP excitation spectrum, which is not susceptible to [Ca^2+^]_i_ variations, can be used to normalize the GCaMP signal [47]. Alternatively, the indicator can be co-expressed together with a fluorescence protein, with which fluorescence signal can be used as a normalizer for movement-linked artifacts [48].

An interesting alternative to single-protein sensors consist of using ratiometric indicators, in which variations of the ratio of the fluorescence emitted by two fluorescent proteins are correlated to [Ca^2+^]_i_. One example of these sensors is the FRET-based cameleon family of probes [49], in which the [Ca^2+^] responsive elements M13 and CaM are bound to a cyan (CFP) and a yellow (YFP) variant of the GFP, respectively. Upon excitation, the CFP emits at 480 nm in the absence of Ca^2+^, while in the presence of Ca^2+^, the interaction between CaM and M13 brings CFP and YFP closer, and the energy released from the CFP is absorbed (and thus emitted at 535 nm) by the YFP. By calculating the ratio of the YFP/CFP fluorescence emissions, it is possible to infer [Ca^2+^]_i_ with no interference from focal plane shifts, photobleaching, or dye leakage.

Efforts for the amelioration of existing GECIs and for the development of new ones are continuously ongoing. This path precedes in parallel with the improvement of imaging instrumentation [50]. Indeed, early Ca^2+^ imaging experiments in live tissues/animals were performed with epifluorescence microscopy equipped with charged coupled detector (CCD)-based cameras using synthetic dyes. However, the application of wide-field microscopy is limited by light scattering across the z-axis and resolution and contrast fall as the imaging plane goes deeper into the tissues. As a result, this technique can only be successful with small and transparent invertebrates [51]. These limitations were overcome by the development of confocal microscopy, which takes advantage of a focused illumination light and a pinhole that rejects scattered out-of-focus light. However, the increase in excitation necessary to compensate the signal loss causes photobleaching and photodamage [52], thus once again limiting the application of confocal imaging to in vitro or very thin preparations [22,53]. The development of two-photon (2P) microscopy [52] allowed the study of Ca^2+^ dynamics within cells localized deeper in tissues. Importantly, this permitted to move from cell cultures and isolated tissues to the imaging of the intact brain of an entire transgenic animal, exploring neuronal circuitry in vivo [51,53,54,55]. The continuous implementation of the system, e.g., the use of gradient refractive index (GRIN) lenses and microendoscopy, recently allowed imaging of deeper subcortical structures in awake mice [56,57,58,59]. Moreover, to overcome the limitation of the restricted field of view of high-resolution microscopy, which is usually smaller than a mouse brain, mesoscale 2P microscopes have been developed, allowing the imaging of extended brain areas with subcellular resolution [60]. Miniaturized, head-mounted microscopes with a large field of view have been also developed in order to expand the activity mapping in the dorsal cortex in freely behaving mice [61]. 

Thanks to this parallel development of fluorescent indicators and dedicated instrumentation, Ca^2+^ imaging has developed into a powerful tool for the imaging of the brain in living animals. As technology advances, upgraded systems could be designed to map less accessible brain areas in order to obtain a larger view of the nervous system with increased spatio-temporal resolution.

## 3. Ca^2+^ Imaging in Mice

Among species, mice are the most common animal models used in research. This is mainly due to their similarity to humans at the genetic and physiology level as well as their relatively short generation time and lifespan, which make them useful for aging studies [62,63]. Moreover, thanks to the advancement of technologies, new genetic tools [64] and a large number of transgenic mouse models of disease, including neurodegenerative disorders (see, e.g., http://www.alzforum.org/research-models (accessed on 17 August 2021)), are now available. 

Since their development, GECIs are the elective probes for ex vivo/in vivo Ca^2+^ imaging in mice. GECIs can be expressed through in utero electroporation [65] or by injecting viral vectors, such as adeno-associated virus (AAV) or lentivirus, which are known to have efficient tissue transduction and a low immunogenicity [66]. However, the delivery of viral vectors presents some limitations, such as invasive surgery and the uncontrolled level, duration, and specificity of transgene expression [67]. Moreover, the restricted spread of the virus can represent a limit in experiments requiring wide-field imaging, such as mesoscale functional imaging, and multiple injections are often required to guarantee the adequate, widespread expression of the indicator [34]. 

To overcome these limitations and to reach a more stable, long-term expression of Ca^2+^ probes, GECI-expressing transgenic mice have been developed [32,33,68,69,70]. These, in combination with the evolution of appropriated optical imaging techniques, i.e., multiphoton or wide-field systems, led in vivo Ca^2+^ imaging studies, especially those investigating processes related to aging and neurodegeneration in old mice, to a rapid improvement.

The most employed GECIs for live imaging in mice are the GCaMPs. In particular, GCaMP6 [32,39] has been used to study Ca^2+^ dynamics in cellular and subcellular compartments. Improved versions of GCaMP6 have been recently generated, i.e., the enhanced version GCaMP7 [46] and the multicolor XCaMPs, characterized by high signal-to-noise ratio and high-frequency spike resolution and optimized for studying complex Ca^2+^ dynamics in vivo [42,71] (Table 1). 

Although commonly used, GCaMPs could present side effects when expressed chronically. Cytotoxicity and abnormalities in brain activity have been reported [72] due to the interference of CaM with the gating and signaling of L-type Ca^2+^ channels (Ca_V_1). To overcome this drawback, a new GCaMP-X variant was generated by protecting Ca^2+^-free CaM to avoid Ca_V_1 perturbation [73]. Alternatively, inducible/tissue-specific expression of Ca^2+^ indicators can be used, e.g., via Cre/LoxP-mediated recombination [33] or by the tetracycline-controlled transactivator (tTA)/tetracycline response element (TRE) system [74]. Modern genetic approaches, such as the use of tightly regulated genomic locus TIGRE [75] improved these strategies [68] and also expanded the repertoire of driver and reporter transgenic mouse lines for in vivo Ca^2+^ imaging [64].

Another limitation of GCaMPs is represented by their excitation emission spectra. Indeed, traditional GCaMPs cannot be used in mice that concomitantly express other GFP-based proteins. Similarly, their simultaneous use with optogenetic tools is prevented, due to the overlap of a GCaMP excitation spectrum with those of light-sentitive ion channels, such as channelrhodopsin (ChR2). To overcome this weakness, improved red-shifted GECIs with a sensitivity comparable to GcaMP6 were generated: mRuby-based jRCaMP1a and jRCaMP1b, mApple-based jRGECO1a [76], and a near-infrared Ca^2+^ sensor iGECI [77]. The new probes allow deep-tissue imaging and dual-colour imaging together with GFP-based probes and the use of optogenetic tools in Ca^2+^ imaging experiments. 

Organelles play a fundamental role in shaping cellular Ca^2+^ signals. Given their importance in physiology and their involvement in the pathogenesis of neurodegenerative diseases [8,78,79,80], specific Ca^2+^ probes have been developed to target them [38,81]. Nowadays, techniques to measure intra-organelle Ca^2+^ at the cellular level are available and are used routinely. However, this type of measurement is still poorly exploited in living animals. 

As far as the mitochondria are concerned, a large number of probes, both chemical [82] and genetically encoded [83,84,85], are available to monitor organelle Ca^2+^ dynamics in cultured cells. Until recently, however, measurements of mitochondrial Ca^2+^ dynamics in vivo were limited to the use of the virus-mediated delivery of GECIs (see above). Mice lines have only been generated for the expression of mitochondria-targeted bioluminescence-based sensors [86,87] which, however, present a limited spatial resolution. Very recently, we reported the generation of a new transgenic mouse line expressing the mitochondria-targeted cameleon Ca^2+^ sensor 4mtD3cpv [35], which would represent a new valuable tool to investigate mitochondrial Ca^2+^ handling in vivo.

Thanks to the tremendous improvement in Ca^2+^ sensor expression in mice and imaging techniques, Ca^2+^ signalling alterations in a variety of diseases have been described [6,88,89]. For example, a number of studies support the notion of a correlation between Ca^2+^ dysregulation and AD [8,90]. AD is the most common neurodegenerative disorder, characterized by cognitive and behavioral impairment that heavily hinders daily activities. Its diagnosis is confirmed only on post-mortem brain tissue by assessing the presence of senile plaques, formed by extracellular deposits of amyloid β (Aβ) peptides, and intracellular neurofibrillary tangles (NFT) of the microtubule-binding protein tau. The most common forms of AD are sporadic (SAD) without any inheritance, whereas only a small percentage is familial (FAD). The genetic causes of FAD are mutations in the amyloid precursor protein (*APP*), presenilin-1 (*PSEN1*), and presenilin-2 (*PSEN2*) genes. In the late 1980s, Ca^2+^ homeostasis alterations were proposed to be responsible for AD-linked neurodegeneration [91]. Indeed, in the following years, experiments performed in cell cultures, ex vivo and in vivo highlighted that Ca^2+^ handling impairment is an early event in AD pathogenesis [12,90].

In this context, the development of several transgenic AD mouse models (https://www.alzforum.org/research-models (accessed on 17 August 2021)), along with new tools for Ca^2+^ imaging, allowed the investigation of Ca^2+^ dynamics at different levels, ranging from in vivo multiphoton imaging of Ca^2+^ dynamics within organelles [92] to Ca^2+^ imaging in awake mice [93,94]. As an example, advanced Ca^2+^ imaging techniques have recently been applied to explore hippocampal activity in a knock-in AD mouse model carrying mutated APP. These mice were crossed with a transgenic mouse line that co-expresses the Ca^2+^ indicator GCaMP7 and a Ca^2+^-insensitive red fluorescent protein (DsRed2) in neurons [95], and two-photon microscopy was applied to image the activity of 1000 neurons in the dorsal hippocampal CA1 region of awake mice. Neuronal activity was detected in the form of Ca^2+^ transients monitoring GCaMP7 fluorescence, while the red fluorescent protein, showing stable fluorescence independent on neuronal activity, was used to precisely align the imaging field during subsequent acquisitions. Ca^2+^ signals were followed together with Aβ deposition for several months while the animals were subjected to specific tasks that allowed the estimation spatial and temporal representations in the hippocampus separately, revealing that place cells were mostly impaired in AD mice, especially in the proximity of Aβ deposition [93].

## 4. Ca^2+^ Imaging in *Drosophila melanogaster*

The fruit fly *Drosophila melanogaster* is an established model organism in scientific research that offers the advantages of fast and robust genetic manipulation techniques, s lack of genetic redundancy compared to vertebrates, and a completely annotated genome since 2000 [96]. Fly handling is procedurally easy and cheap, and macroscopic phenotypic features allow for the quick selection of individuals [97]. *Drosophila* life cycle starts with the laying of eggs that develop into embryo and then into larvae, which after passing three instar stages undergo pupation followed by metamorphosis, eclose in the adult form. A single fly can lay hundreds of eggs, and the whole cycle lasts about 10 days at 25 °C, making it relatively easy to obtain a large number of experimental individuals. 

The lack of genetic redundancy and the easy genetic manipulation via well-consolidated knock-down and knock-out strategies make the characterization of protein function in *Drosophila* straightforward. Noteworthy, relevant genes encoding Ca^2+^ toolkit players and conserved Ca^2+^ signaling pathways were first described in flies, e.g., the first member of Transient Receptor Potential (TRP) channel superfamily [98] and the Store-Operated Ca^2+^ entry (SOCE) molecular players stromal interacting molecule 1 (STIM1) and ORAi1 [99,100]. Indeed, the basic building blocks of the Ca^2+^ signaling toolkit are conserved between mammals and flies [101]. Single homologs of key molecular players are present in *Drosophila*, such as voltage- and ligand-gated PM Ca^2+^ channels [102], the Ca^2+^ release channels the inositol 1,4,5-trisphosphate receptor (IP_3_R, named itpr) [103] and the Ryanodine Receptor (RyR) [104,105], the Ca^2+^ ATPases located on PM (PMCA) [106], Sarco/ER (SERCA) [107], the secretory pathway membranes (SPCA, named SPoCk) [108], the Na^+^/Ca^2+^ exchanger NCX (Calx) [109], Ca^2+^ interacting proteins, e.g., Calcineurin (CaN), Calmodulin (Cam), Ca^2+^/Calmodulin-dependent protein kinase II (CaMKII), the and Ca^2+^/Calmodulin-dependent serine protein kinase (CaSK).

The ease of transgenesis in *Drosophila* allowed the generation of several lines for the expression of GECIs. Most frequently, the expression of indicators relies on the Gal4-UAS binary expression system [110,111] as follows: One fly strain carries the cDNA coding for the Ca^2+^ sensor under the control of an upstream activating sequence (UAS) so that the gene is silent in the absence of the transcription factor Gal4. A second fly strain expresses Gal4 under the control of a cell specific promoter. Upon mating, the resulting progeny will express the probe in the specific cell type determined by the Gal4 line promoter. UAS lines for the controlled expression of both cytosolic and organelle-targeted GECIs have now been developed in flies and exploited to unravel questions of physiological and pathological relevance.

Although the anatomic divergence between the fruit fly and humans is evident, the *Drosophila* nervous system proved particularly helpful in the study of neuronal function in physiology and pathology. In the adult fly, both a central and a peripheral nervous system can be recognized. The central brain and the optic lobes are lodged in the head capsule. Neurons from the brain descend in the ventral nerve cord, and motor neurons project towards muscles (for a complete description of *Drosophila* nervous system anatomy, the reader can refer to [112]). The adult fly brain comprises about 200,000 neurons [113]. In the central brain alone, over 20 million connections have been mapped [114]. Similar to mammals, these neurons are organized in distinct, highly interconnected regions, indicated as neuropils, that are able to mediate complex behaviors, such as learning, smell and vision, circadian rhythms, courtship and mating, aerial navigation, and memory [115,116]. Importantly, as is also the case for humans, behavioral performance in flies decline with age [117]. Moreover, the molecular mechanisms that govern neuronal functionality, including axon pathfinding, synaptogenesis, membrane excitability, and neurotransmission are conserved between *Drosophila* and mammals [16]. 

From the experimental point of view, the great advantage of using *Drosophila* as a model organism is the high accessibility of its nervous system at all developmental stages. Ca^2+^ imaging procedures have been adapted or specifically developed for the study of Ca^2+^ dynamics in vivo in the nervous system in embryos [118,119], larvae [120,121,122,123], and also in adult flies [124]. A critical issue is to obtain adequate excitation light access to the structure of interest. *Drosophila* larvae are partially transparent, and the GECIs expressed in superficial tissues, such as central nervous system (CNS) or sensory neurons, can be excited through the intact animal’s cuticle, provided that the fluorescence is strong enough. The main obstacle to larval Ca^2+^ imaging is the sample movement due to continuous body wall muscle contraction, which can be partially avoided using sticky tape [125] or chambers [126] and corrected by using ratiometric GECIs (see above). Adult CNS can be imaged across the cuticle using 3P microscopy [127] or, most frequently, accessed upon surgery [125,128]. Single-wavelength GECIs, such as GCaMPs, are mostly used in adult brains since these preparations are less subjected to movement-linked artefacts. The ease of genetic manipulation in flies allows the combination of Ca^2+^ imaging with other genetically encoded tools, e.g., optogenetics [129,130,131] and genetically encoded voltage indicators (GEVIs) [132,133]. GECIs expressed in flies can also be exploited to perform in vitro, i.e., primary neuronal culture [26,27,134], or ex vivo Ca^2+^ imaging experiments, e.g., larval neuromuscular preparation [36] or the isolated larval brain [135]. 

Optical Ca^2+^ imaging techniques can be applied to explore a wide range of biologically relevant questions in *Drosophila* [136]. Mechanisms of sensory processing have been extensively explored in flies using Ca^2+^ imaging techniques to investigate the response of specific brain regions to various sensory stimuli. These studies mainly investigated olfaction [137,138,139,140,141,142], visual circuits [143], auditory stimuli [144], taste [145], mechanosensation [146], and thermosensation [125]. Moreover, Ca^2+^ imaging using GECIs has been successfully applied to the *Drosophila* brain to investigate the neuronal plasticity underlying associative learning and memory formation [139] and the large-scale mapping of functional brain connectivity [147]. Whole-brain imaging during adult fly open field behavior represents an interesting task that would allow Ca^2+^ imaging to be performed in freely walking flies during sensorial and social behaviors [148]. Long-term (>24 h) chronic preparations, which are fundamental to dissect the mechanisms of plasticity, neurodegeneration, and aging, can be exploited, although they are quite critical from the experimental point of view due to fly fragility and their opaque exoskeleton [128].

The potential of the described approaches makes *Drosophila* a powerful model system to elucidate the Ca^2+^ dynamics involved in pathogenic processes in neurodegenerative diseases. Remarkably, over 60% of known human disease-causing genes have a fly homolog [149], and a wide collection of fly models of neurodegenerative disorders have been developed so far. Disease models have been created in flies by expressing human disease-associated proteins (or their fly homologs). In the case of loss of function disease mutations, knock out/knock down approaches have also proved successful in mimicking the pathology. Neurodegeneration has been observed in many of these models, confirming their reliability.

As an example, *Drosophila* AD models have been generated, and neuronal Ca^2+^ homeostasis has been evaluated using different approaches, e.g., loading the chemical indicator fura-2 in dissociated neurons [150,151] or imaging the whole brain of adult flies expressing GECIs ex vivo [152] or in vivo through a cranial window [153]. AD fly models expressing mitochondria-targeted GECIs were also exploited, and mitochondrial Ca^2+^ levels have been measured in dissected third instar larval brains [154]. 

## 5. Ca^2+^ Imaging in Zebrafish

Zebrafish (*Danio rerio*) is a small freshwater teleost native of Southeast Asia belonging to the cyprinid family. In recent years, it has become a prominent vertebrate model in neuroscience as well as in other fields of biological research. The reasons for its increasing popularity stem from characteristics that allow it to overcome many limitations that emerge when working with other vertebrates, especially tissue opacity and the large size and complexity of the brain. Given its small size, rapid development (precursors of the internal organs appear by 36 h post fertilization) and the short generation time (generally 3–4 months), zebrafish are easy to manipulate and to raise in the laboratory [155]. 

Notably, the zebrafish genome has been completely sequenced. Interestingly, 70% of zebrafish genes share homology with the human genome, and 84% of genes associated with diseases in humans are present in zebrafish [14,15]. Moreover, a large homology in brain structure and neurochemistry is present between zebrafish and the other vertebrates [156], making e the investigation of neuronal physiology and pathology possible.

Despite the small size of the zebrafish brain (~300 μm thick, 400 μm wide, and 800 μm long), it maintains the basic vertebrate brain organization, although at a lower-level complexity. Moreover, the transparency of the embryos and larvae makes the entire nervous system optically accessible, allowing the brain-wide monitoring of neuronal activity throughout the whole CNS in real time and at single-cell resolution [157,158,159,160,161,162,163]. A total of 100,000 neurons have been counted in larval CNS, which is relatively simple yet capable of handling a wide array of activities, e.g., visually guided behaviors such as optomotor and optokinetic response, phototaxis, and prey capture [164,165,166,167]. Altogether, these characteristics make zebrafish larvae powerful models to study the neuronal mechanisms underlying complex behaviors at the synaptic, cellular, and circuit level [162].

Zebrafish disease models can be generated in order to explore the progression of pathologies and the alterations of related signaling pathways in vivo [168,169,170,171]. Reverse genetic techniques can be employed to transiently manipulate target genes, e.g., by mRNA or morpholino antisense oligonucleotide injection into fertilized eggs at one- to four-cell stage to induce the overexpression or knock down of specific disease-associated proteins [171,172] or by using most recent genome editing techniques such as CRISPR-Cas9 [173]. Notably, due to the low-cost availability of large samples, zebrafish are also amenable for high-throughput screening of large compound libraries [174,175], allowing the very effective selection of new drug candidates without the costly and time-consuming processes linked to targeted drug design. 

One of the great advantages of zebrafish is the ease of application of techniques ranging from advanced Ca^2+^ imaging, electrophysiological recordings, and optogenetics, allowing the investigation of neuronal activity in vivo at single cell resolution as well as full circuit reconstruction. 

Dextran-conjugated and membrane-permeant chemical indicators have been employed to image Ca^2+^ in zebrafish. However, they require injection procedures and do not allow the selective loading of specific cell types. For these reasons, the GECIs represent the elective choice for Ca^2+^ imaging experiments in zebrafish. Transgenic lines can be generated by microinjecting [176] the GECI cDNA flanked by tol2 recombination recognition sites into fertilized eggs (at one-cell stage) together with Tol2 transcript. This results in the random recombination of the transgene with genomic DNA [177]. Transgenes are usually placed under the control of cell-specific gene enhancers or promoters, e.g., HuC promoter (referred to as elavl3), which labels the whole nervous system, to guarantee GECI expression in specific cell types or at a specific time during development. 

GECIs, such as aequorin, cameleons, and GCaMPs, have been exploited in zebrafish, allowing Ca^2+^ imaging experiments to be performed in intact, free moving animals [23,178]. Neuronal activity can be evaluated in specific cellular types in which the reporter is expressed [44,157,158,159,160,161] in response to multiple stimuli and/or conditions. The advantage of expressing fluorescent Ca^2+^ sensors in specific cell types, in combination with appropriate imaging techniques (i.e., confocal, 2P, light-sheet microscopy), is that it allows to image neuron functional dynamics at early stages of zebrafish development as well as during neurodegeneration. As a result, a morphological and functional reconstruction of the pathways underlying brain function and dysfunction can be obtained with a high spatial and temporal resolution, a task that is currently only achievable in transparent zebrafish larva. 

## 6. Ca^2+^ Imaging in *Caenorhabditis elegans*

*Caenorhabditis elegans* is a nonparasitic nematode firstly described as a model organism in 1963. Only a decade later, it was clear that worms could be used as a genetic tool to study aging and neurodegeneration [179]. The success of this model organism is linked to some of its experimental highlights, including (i) their short lifespan, around 20 days; (ii) their large homogeneous progeny, as hermaphrodites, they normally reproduce by self-fertilization; (iii) their simple anatomy, which still includes nervous, muscular, digestive, and reproductive systems [180]. Moreover, the *C. elegans* genome was completely sequenced in 1998 [181], and posterior genomic and proteomic comparative analysis showed that around 83% of the worm proteome has predicted human homologous genes [182,183]. 

The *C. elegans* nervous system is extremely simple when compared to mammals yet complex enough to consider this model suitable to study neurodegeneration. Notably, the full connectome of the 302 neurons comprising the hermaphrodite worm nervous system has been mapped [184,185,186]. Despite the reduced number of neurons, *C. elegans* presents standard glutamatergic [187], GABAergic [188], cholinergic [189], dopaminergic [190], and serotoninergic [191] transmission, and subpopulations of sensory neurons, interneurons, and motor-neurons have been identified. 

The availability of the full genomic sequence of *C. elegans* led to the identification of homologs of Ca^2+^ signalling components, including PM [192,193] and intracellular Ca^2+^ channels, such as IP_3_R [194] and RyR [195,196]; the Ca^2+^ pumps SERCA [197] and PMCA [198]; transporters, such as NCX [199]; and SOCE molecular players [200]. Furthermore, a plethora Ca^2+^ signalling modulators are present in *C. elegans,* including CaM, kinases, and phosphatases [201,202,203].

One of the features that make *C. elegans* an advantageous animal model is its genetic manipulability. Forward and reverse genetic approaches have been largely exploited in worms, and a considerable number of transgenic lines have been generated over time [204,205]. Moreover, the availability of a genome-wide RNAi library [206] allows reverse genetics screening. The approach is particularly relevant since it provides partial downregulation of the expression of proteins whose complete loss would result in early lethality [197,207]. Pharmacological high throughput screenings can also be performed in *C. elegans* models of neurodegenerative diseases to search for small molecules as possible therapeutic treatments [208,209].

Consistently, *C. elegans* models carrying gain/loss of function mutations of genes involved in neurodegenerative diseases have been generated, recapitulating many of the characteristics of the disease at the cellular level [210,211]. These models provide interesting possibilities for the study of disease mechanisms.

Ca^2+^ sensors can be expressed in *C. elegans* in a tissue specific manner. A fundamental feature for Ca^2+^ imaging experiments is the transparency of the worms throughout their lives. It is thus possible to record Ca^2+^ sensor fluorescence in vivo in the intact animal. The two most broadly used Ca^2+^ sensors in *C. elegans* are GCaMPs and FRET-based cameleons, the most frequently expressed in neurons or muscular cells [44,212,213,214], alone or under the optogenetic manipulation of neuronal activity [215]. Although the majority of the studies in *C. elegans* have been performed using untargeted probes expressed in the cytosol, relevant examples of Ca^2+^ sensors expressed in organelles exist, e.g., mitochondria [216,217,218]. Recently, advanced approaches allowed whole-brain Ca^2+^ imaging, in which about 100 neurons were simultaneously monitored in moving worms [219,220].

The application of these powerful techniques to *C. elegans* models have provided critical information on the role of Ca^2+^ signaling in aging [221]. As an example, the dynamics of cytosolic and mitochondrial Ca^2+^ during aging have been recorded in live, non-anesthetized worms [216,222].

## 7. Final Remarks

Here, we depicted four commonly used animal models used to study neurodegeneration, highlighting their characteristics and potentiality in the field of Ca^2+^ imaging. The combination of genetic accessibility and methodological advances has enabled the exploration of Ca^2+^ dynamics not only inside single cells but also among a population of neurons. As a result, cellular and circuit neurophysiology can be explored in physio-pathological conditions.

Many relevant examples of mouse and *Drosophila* models of neurodegenerative diseases exist where Ca^2+^ dynamics have been successfully explored. Additionally, Ca^2+^ probes can be expressed in *C. elegans* and zebrafish, two models that have great potential in terms of genetic manipulation. We foresee that these organisms will be profitably used to explore Ca^2+^ dynamics in models of neurodegenerative disorders [223,224].

Thanks to the improvement of both Ca^2+^ sensors and imaging instrumentation, the study of Ca^2+^ dynamics is progressively moving from in vitro samples to animals in vivo, providing an ever-growing number of strategies for investigating neural function and dysfunction. It is however worth to mention that in recent years, three-dimensional (3D) brain organoids from patient-derived, inducible pluripotent stem cells (iPSCs) have been developed, showing themselves to be useful models for studying different aspects of aging and neurodegeneration [225,226,227].

## Figures and Tables

**Table 1:** Examples of the most common GCaMP sensors used for Ca^2+^ imaging and relative affinity for Ca^2+^ (K_d_).

	Reference	Characteristics	Variants	K_d_ (nM)
**GCaMP3**	Tian et al., 2009 [40]	Adequate fluorescence at resting cell [Ca^2+^] for the detection of expressing cells. Unable to detect single action potentials (APs) in vivo.	GCaMP3	345 ± 17 [43]
**GCaMP5**	Akerboom et al., 2012 [44]	Improved dynamic range (triplicated in GCaMP5G, compared to GCaMP3). Variants can be chosen according to their properties (e.g., affinity for Ca^2+^) to fit particular experimental requirements.	GCaMP5A	307 ± 12
			GCaMP5D	730 ± 18
			GCaMP5G	460 ± 11
			GCaMP5K	189 ± 5
			GCaMP5L	390 ± 18
**GCaMP6**	Chen et al., 2013 [45]	Able to detect single APs (GCaMP6f presents the shortest half-decay time). Variants can be chosen according to their properties (e.g., affinity for Ca^2+^) to fit particular experimental requirements. Used to image large groups of neurons, as well as tiny synaptic compartments. Adequate for imaging experiments over multiple weeks. Adequate fluorescence at basal cell [Ca^2+^].	GCaMP6s	144 ± 4
			GCaMP6m	167 ± 3
			GCaMP6f	375 ± 14
**jGCaMP7**	Dana et al., 2019 [46]	Enhanced signal-to-noise ratio allows detection of individual spikes over wide fields of view (jGCaMP7s,f). Used for imaging of small processes thanks to the high brightness (jGCaMP7b).	jGCaMP7s	68 ± 5
			jGCaMP7f	174 ± 9
			jGCaMP7c	298 ± 5
			jGCaMP7b	82 ± 6
**XCaMP**	Inoue et al., 2019 [42]	Multiple color variants (blue, green, yellow, red). Improved signal linearity. Adequate fluorescence at basal cell [Ca^2+^] (XCaMP-G). Enables measurement of fast-spike trains in individual neurons.	XCaMP-G	200 ± 8
			XCaMP-Gfo	128 ± 5
			XCaMP-Gf	115 ± 4
			XCaMP-R	97 ± 10
			XCaMP-Y	81 ± 6
			XCaMP-B	71 ± 3

## References

[1] Berridge M.J. (1998). Neuronal calcium signaling. Neuron.

[2] Berridge M.J., Bootman M.D., Roderick H.L. (2003). Calcium signalling: Dynamics, homeostasis and remodelling. Nat. Rev. Mol. Cell Biol..

[3] Neher E., Sakaba T. (2008). Multiple roles of calcium ions in the regulation of neurotransmitter release. Neuron.

[4] Zucker R.S. (1999). Calcium- and activity-dependent synaptic plasticity. Curr. Opin. Neurobiol..

[5] Greer P.L., Greenberg M.E. (2008). From Synapse to Nucleus: Calcium-Dependent Gene Transcription in the Control of Synapse Development and Function. Neuron.

[6] Schrank S., Barrington N., Stutzmann G.E. (2020). Calcium-handling defects and neurodegenerative disease. Cold Spring Harb. Perspect. Biol..

[7] Zampese E., Surmeier D.J. (2020). Calcium, Bioenergetics, and Parkinson’s Disease. Cells.

[8] Galla L., Redolfi N., Pozzan T., Pizzo P., Greotti E. (2020). Intracellular Calcium Dysregulation by the Alzheimer’s Disease-Linked Protein Presenilin 2. Int. J. Mol. Sci..

[9] Ureshino R.P., Erustes A.G., Bassani T.B., Wachilewski P., Guarache G.C., Nascimento A.C., Costa A.J., Smaili S.S., da Silva Pereira G.J. (2019). The Interplay between Ca^2+^ Signaling Pathways and Neurodegeneration. Int. J. Mol. Sci..

[10] Surmeier D.J., Schumacker P.T., Guzman J.D., Ilijic E., Yang B., Zampese E. (2017). Calcium and Parkinson’s disease. Biochem. Biophys. Res. Commun..

[11] Bezprozvanny I., Hayden M.R. (2004). Deranged neuronal calcium signaling and Huntington disease. Biochem. Biophys. Res. Commun..

[12] Agostini M., Fasolato C. (2016). When, where and how? Focus on neuronal calcium dysfunctions in Alzheimer’s Disease. Cell Calcium.

[13] Pchitskaya E., Popugaeva E., Bezprozvanny I. (2018). Calcium signaling and molecular mechanisms underlying neurodegenerative diseases. Cell Calcium.

[14] Barbazuk W.B., Korf I., Kadavi C., Heyen J., Tate S., Wun E., Bedell J.A., McPherson J.D., Johnson S.L. (2000). The syntenic relationship of the zebrafish and human genomes. Genome Res..

[15] Howe K., Clark M.D., Torroja C.F., Torrance J., Berthelot C., Muffato M., Collins J.E., Humphray S., McLaren K., Matthews L. (2013). The zebrafish reference genome sequence and its relationship to the human genome. Nature.

[16] Yoshihara M., Ensminger A.W., Littleton J.T. (2001). Neurobiology and the Drosophila genome. Funct. Integr. Genom..

[17] Greek R., Rice M.J. (2012). Animal models and conserved processes. Theor. Biol. Med. Model..

[18] Tsien R.Y. (1981). A non-disruptive technique for loading calcium buffers and indicators into cells. Nature.

[19] Grynkiewicz G., Poenie M., Tsien R.Y. (1985). A new generation of Ca^2+^ indicators with greatly improved fluorescence properties. J. Biol. Chem..

[20] Miyawaki A., Llopis J., Heim R., McCaffery J.M., Adams J.A., Ikura M., Tsien R.Y. (1997). Fluorescent indicators for Ca^2+^ based on green fluorescent proteins and calmodulin. Nature.

[21] Vanni M.P., Chan A.W., Balbi M., Silasi G., Murphy T.H. (2017). Mesoscale Mapping of Mouse Cortex Reveals Frequency-Dependent Cycling between Distinct Macroscale Functional Modules. J. Neurosci..

[22] Grienberger C., Konnerth A. (2012). Imaging Calcium in Neurons. Neuron.

[23] Mao T., O’Connor D.H., Scheuss V., Nakai J., Svoboda K. (2008). Characterization and subcellular targeting of GCaMP-type genetically-encoded calcium indicators. PLoS ONE.

[24] Greotti E., De Stefani D. (2020). Biosensors for detection of calcium. Methods Cell Biol..

[25] Kipanyula M.J., Contreras L., Zampese E., Lazzari C., Wong A.K.C., Pizzo P., Fasolato C., Pozzan T. (2012). Ca^2+^ dysregulation in neurons from transgenic mice expressing mutant presenilin 2. Aging Cell.

[26] Vajente N., Norante R., Redolfi N., Daga A., Pizzo P., Pendin D. (2019). Microtubules Stabilization by Mutant Spastin Affects ER Morphology and Ca^2+^ Handling. Front. Physiol..

[27] Venkiteswaran G., Hasan G. (2009). Intracellular Ca^2+^ signaling and store-operated Ca^2+^ entry are required in Drosophila neurons for flight. Proc. Natl. Acad. Sci. USA.

[28] Borst A., Egelhaaf M. (1992). In vivo imaging of calcium accumulation in fly interneurons as elicited by visual motion stimulation. Proc. Natl. Acad. Sci. USA.

[29] Jain P.T., Chang S.H., Gutry P.P., Berezesky I.K., Trump B.F. (1993). The Relationship Between [Ca^2+^]_i_ and Cell Death Using an *In Vivo* Model: A Study Using the ced-1 Mutant Strain of *C. elegans*. Toxicol. Pathol..

[30] Macleod G.T., Hegström-Wojtowicz M., Charlton M.P., Atwood H.L. (2002). Fast Calcium Signals in *Drosophila* Motor Neuron Terminals. J. Neurophysiol..

[31] Karunanithi S., Georgiou J., Charlton M.P., Atwood H.L. (1997). Imaging of Calcium in *Drosophila* Larval Motor Nerve Terminals. J. Neurophysiol..

[32] Dana H., Chen T.-W., Hu A., Shields B.C., Guo C., Looger L.L., Kim D.S., Svoboda K. (2014). Thy1-GCaMP6 Transgenic Mice for Neuronal Population Imaging In Vivo. PLoS ONE.

[33] Zariwala H.A., Borghuis B.G., Hoogland T.M., Madisen L., Tian L., De Zeeuw C.I., Zeng H., Looger L.L., Svoboda K., Chen T.-W. (2012). A Cre-Dependent GCaMP3 Reporter Mouse for Neuronal Imaging In Vivo. J. Neurosci..

[34] Michelson N.J., Vanni M.P., Murphy T.H. (2019). Comparison between transgenic and AAV-PHP.eB-mediated expression of GCaMP6s using in vivo wide-field functional imaging of brain activity. Neurophotonics.

[35] Redolfi N., Greotti E., Zanetti G., Hochepied T., Fasolato C., Pendin D., Pozzan T. (2021). A New Transgenic Mouse Line for Imaging Mitochondrial Calcium Signals. Function.

[36] Oliva M.K., Pérez-Moreno J.J., O’Shaughnessy J., Wardill T.J., O’Kane C.J. (2020). Endoplasmic Reticulum Lumenal Indicators in Drosophila Reveal Effects of HSP-Related Mutations on Endoplasmic Reticulum Calcium Dynamics. Front. Neurosci..

[37] Li H., Wang X., Zhang N., Gottipati M.K., Parpura V., Ding S. (2014). Imaging of mitochondrial Ca^2+^ dynamics in astrocytes using cell-specific mitochondria-targeted GCaMP5G/6s: Mitochondrial Ca^2+^ uptake and cytosolic Ca^2+^ availability via the endoplasmic reticulum store. Cell Calcium.

[38] Pendin D., Greotti E., Lefkimmiatis K., Pozzan T. (2017). Exploring cells with targeted biosensors. J. Gen. Physiol..

[39] Ding J., Luo A.F., Hu L., Wang D., Shao F. (2014). Structural basis of the ultrasensitive calcium indicator GCaMP6. Sci. China Life Sci..

[40] Tian L., Hires S.A., Mao T., Huber D., Chiappe M.E., Chalasani S.H., Petreanu L., Akerboom J., McKinney S.A., Schreiter E.R. (2009). Imaging neural activity in worms, flies and mice with improved GCaMP calcium indicators. Nat. Methods.

[41] Nakai J., Ohkura M., Imoto K. (2001). A high signal-to-noise Ca^2+^ probe composed of a single green fluorescent protein. Nat. Biotechnol..

[42] Inoue M., Takeuchi A., Manita S., Horigane S., Sakamoto M., Kawakami R., Yamaguchi K., Otomo K., Yokoyama H., Kim R. (2019). Rational Engineering of XCaMPs, a Multicolor GECI Suite for In Vivo Imaging of Complex Brain Circuit Dynamics. Cell.

[43] Chen Y., Song X., Ye S., Miao L., Zhu Y., Zhang R.G., Ji G. (2013). Structural insight into enhanced calcium indicator GCaMP3 and GCaMPJ to promote further improvement. Protein Cell.

[44] Akerboom J., Chen T.W., Wardill T.J., Tian L., Marvin J.S., Mutlu S., Calderón N.C., Esposti F., Borghuis B.G., Sun X.R. (2012). Optimization of a GCaMP calcium indicator for neural activity imaging. J. Neurosci..

[45] Chen T.W., Wardill T.J., Sun Y., Pulver S.R., Renninger S.L., Baohan A., Schreiter E.R., Kerr R.A., Orger M.B., Jayaraman V. (2013). Ultrasensitive fluorescent proteins for imaging neuronal activity. Nature.

[46] Dana H., Sun Y., Mohar B., Hulse B.K., Kerlin A.M., Hasseman J.P., Tsegaye G., Tsang A., Wong A., Patel R. (2019). High-performance calcium sensors for imaging activity in neuronal populations and microcompartments. Nat. Methods.

[47] Tosatto A., Sommaggio R., Kummerow C., Bentham R.B., Blacker T.S., Berecz T., Duchen M.R., Rosato A., Bogeski I., Szabadkai G. (2016). The mitochondrial calcium uniporter regulates breast cancer progression via HIF-1α. EMBO Mol. Med..

[48] Berry J.A., Cervantes-Sandoval I., Chakraborty M., Davis R.L. (2015). Sleep Facilitates Memory by Blocking Dopamine Neuron-Mediated Forgetting. Cell.

[49] Váradi A., Rutter G.A. (2002). Green fluorescent protein calcium biosensors. Calcium imaging with GFP cameleons. Methods Mol. Biol..

[50] Sancataldo G., Silvestri L., Allegra Mascaro A.L., Sacconi L., Pavone F.S. (2019). Advanced fluorescence microscopy for in vivo imaging of neuronal activity. Optica.

[51] Russell J.T. (2011). Imaging calcium signals *in vivo*: A powerful tool in physiology and pharmacology. Br. J. Pharmacol..

[52] Denk W., Svoboda K. (1997). Photon Upmanship: Why Multiphoton Imaging Is More than a Gimmick. Neuron.

[53] Svoboda K., Yasuda R. (2006). Principles of two-photon excitation microscopy and its applications to neuroscience. Neuron.

[54] Stosiek C., Garaschuk O., Holthoff K., Konnerth A. (2003). In vivo two-photon calcium imaging of neuronal networks. Proc. Natl. Acad. Sci. USA.

[55] Brondi M., Moroni M., Vecchia D., Molano-Mazón M., Panzeri S., Fellin T. (2020). High-Accuracy Detection of Neuronal Ensemble Activity in Two-Photon Functional Microscopy Using Smart Line Scanning. Cell Rep..

[56] Qin Z., Chen C., He S., Wang Y., Tam K.F., Ip N.Y., Qu J.Y. (2020). Adaptive optics two-photon endomicroscopy enables deep-brain imaging at synaptic resolution over large volumes. Sci. Adv..

[57] Meng G., Liang Y., Sarsfield S., Jiang W., Lu R., Dudman J.T., Aponte Y., Ji N. (2019). High-throughput synapse-resolving two-photon fluorescence microendoscopy for deep-brain volumetric imaging in vivo. eLife.

[58] Chien Y.-F., Lin J.-Y., Yeh P.-T., Hsu K.-J., Tsai Y.-H., Chen S.-K., Chu S.-W. (2021). Dual GRIN lens two-photon endoscopy for high-speed volumetric and deep brain imaging. Biomed. Opt. Express.

[59] Antonini A., Sattin A., Moroni M., Bovetti S., Moretti C., Succol F., Forli A., Vecchia D., Rajamanickam V.P., Bertoncini A. (2020). Extended field-of-view ultrathin microendoscopes for high-resolution two-photon imaging with minimal invasiveness. eLife.

[60] Sofroniew N.J., Flickinger D., King J., Svoboda K. (2016). A large field of view two-photon mesoscope with subcellular resolution for in vivo imaging. eLife.

[61] Rynes M.L., Surinach D.A., Linn S., Laroque M., Rajendran V., Dominguez J., Hadjistamoulou O., Navabi Z.S., Ghanbari L., Johnson G.W. (2021). Miniaturized head-mounted microscope for whole-cortex mesoscale imaging in freely behaving mice. Nat. Methods.

[62] Rosenthal N., Brown S. (2007). The mouse ascending: Perspectives for human-disease models. Nat. Cell Biol..

[63] Perlman R.L. (2016). Mouse Models of Human Disease: An Evolutionary Perspective. Evol. Med. Public Health.

[64] Daigle T.L., Madisen L., Hage T.A., Valley M.T., Knoblich U., Larsen R.S., Takeno M.M., Huang L., Gu H., Larsen R. (2018). A Suite of Transgenic Driver and Reporter Mouse Lines with Enhanced Brain-Cell-Type Targeting and Functionality. Cell.

[65] Gee J.M., Gibbons M.B., Taheri M., Palumbos S., Morris S.C., Smeal R.M., Flynn K.F., Economo M.N., Cizek C.G., Capecchi M.R. (2015). Imaging activity in astrocytes and neurons with genetically encoded calcium indicators following in utero electroporation. Front. Mol. Neurosci..

[66] Maes M.E., Colombo G., Schulz R., Siegert S. (2019). Targeting microglia with lentivirus and AAV: Recent advances and remaining challenges. Neurosci. Lett..

[67] Davidson B.L., Breakefield X.O. (2003). Viral vectors for gene delivery to the nervous system. Nat. Rev. Neurosci..

[68] Madisen L., Garner A.R., Shimaoka D., Chuong A.S., Klapoetke N.C., Li L., van der Bourg A., Niino Y., Egolf L., Monetti C. (2015). Transgenic Mice for Intersectional Targeting of Neural Sensors and Effectors with High Specificity and Performance. Neuron.

[69] Wekselblatt J.B., Flister E.D., Piscopo D.M., Niell C.M. (2016). Large-scale imaging of cortical dynamics during sensory perception and behavior. J. Neurophysiol..

[70] Agarwal A., Wu P.-H., Hughes E.G., Fukaya M., Tischfield M.A., Langseth A.J., Wirtz D., Bergles D.E. (2017). Transient Opening of the Mitochondrial Permeability Transition Pore Induces Microdomain Calcium Transients in Astrocyte Processes. Neuron.

[71] Inoue M. (2021). Genetically encoded calcium indicators to probe complex brain circuit dynamics in vivo. Neurosci. Res..

[72] Steinmetz N.A., Buetfering C., Lecoq J., Lee C.R., Peters A.J., Jacobs E.A.K., Coen P., Ollerenshaw D.R., Valley M.T., de Vries S.E.J. (2017). Aberrant Cortical Activity in Multiple GCaMP6-Expressing Transgenic Mouse Lines. eNeuro.

[73] Yang Y., Liu N., He Y., Liu Y., Ge L., Zou L., Song S., Xiong W., Liu X. (2018). Improved calcium sensor GCaMP-X overcomes the calcium channel perturbations induced by the calmodulin in GCaMP. Nat. Commun..

[74] Sato M., Kawano M., Ohkura M., Gengyo-Ando K., Nakai J., Hayashi Y. (2015). Generation and Imaging of Transgenic Mice that Express G-CaMP7 under a Tetracycline Response Element. PLoS ONE.

[75] Zeng H., Horie K., Madisen L., Pavlova M.N., Gragerova G., Rohde A.D., Schimpf B.A., Liang Y., Ojala E., Kramer F. (2008). An Inducible and Reversible Mouse Genetic Rescue System. PLoS Genet..

[76] Dana H., Mohar B., Sun Y., Narayan S., Gordus A., Hasseman J.P., Tsegaye G., Holt G.T., Hu A., Walpita D. (2016). Sensitive red protein calcium indicators for imaging neural activity. eLife.

[77] Shemetov A.A., Monakhov M.V., Zhang Q., Canton-Josh J.E., Kumar M., Chen M., Matlashov M.E., Li X., Yang W., Nie L. (2021). A near-infrared genetically encoded calcium indicator for in vivo imaging. Nat. Biotechnol..

[78] Zampese E., Pizzo P. (2012). Intracellular organelles in the saga of Ca^2+^ homeostasis: Different molecules for different purposes?. Cell. Mol. Life Sci..

[79] Stanga S., Caretto A., Boido M., Vercelli A. (2020). Mitochondrial dysfunctions: A red thread across neurodegenerative diseases. Int. J. Mol. Sci..

[80] Trinh D., Israwi A.R., Arathoon L.R., Gleave J.A., Nash J.E. (2021). The multi-faceted role of mitochondria in the pathology of Parkinson’s disease. J. Neurochem..

[81] Suzuki J., Kanemaru K., Iino M. (2016). Genetically Encoded Fluorescent Indicators for Organellar Calcium Imaging. Biophys. J..

[82] Pendin D., Norante R., De Nadai A., Gherardi G., Vajente N., Basso E., Kaludercic N., Mammucari C., Paradisi C., Pozzan T. (2019). A Synthetic Fluorescent Mitochondria-Targeted Sensor for Ratiometric Imaging of Calcium in Live Cells. Angew. Chem. Int. Ed..

[83] Rizzuto R., Simpson A.W., Brini M., Pozzan T. (1992). Rapid changes of mitochondrial Ca^2+^ revealed by specifically targeted recombinant aequorin. Nature.

[84] Palmer A.E., Giacomello M., Kortemme T., Hires S.A., Lev-Ram V., Baker D., Tsien R.Y. (2006). Ca^2+^ Indicators Based on Computationally Redesigned Calmodulin-Peptide Pairs. Chem. Biol..

[85] Pendin D., Greotti E., Filadi R., Pozzan T. (2015). Spying on organelle Ca^2+^ in living cells: The mitochondrial point of view. J. Endocrinol. Investig..

[86] Rogers K.L., Picaud S., Roncali E., Boisgard R., Colasante C., Stinnakre J., Tavitian B., Brulet P. (2007). Non-invasive in vivo imaging of calcium signaling in mice. PLoS ONE.

[87] Cainarca S., Fenu S., Ferri C., Nucci C., Arioli P., Menegon A., Piemonti L., Lohmer S., Wrabetz L., Corazza S. (2010). A Photoprotein in Mouse Embryonic Stem Cells Measures Ca^2+^ Mobilization in Cells and in Animals. PLoS ONE.

[88] Petersen O.H., Gerasimenko O.V., Gerasimenko J.V. (2011). Pathobiology of acute pancreatitis: Focus on intracellular calcium and calmodulin. F1000 Med. Rep..

[89] Marchi S., Giorgi C., Galluzzi L., Pinton P. (2020). Ca^2+^ Fluxes and Cancer. Mol. Cell.

[90] Pizzo P., Basso E., Filadi R., Greotti E., Leparulo A., Pendin D., Redolfi N., Rossini M., Vajente N., Pozzan T. (2020). Presenilin-2 and Calcium Handling: Molecules, Organelles, Cells and Brain Networks. Cells.

[91] Khachaturian Z.S. (1987). Hypothesis on the regulation of cytosol calcium concentration and the aging brain. Neurobiol. Aging.

[92] Calvo-Rodriguez M., Hou S.S., Snyder A.C., Kharitonova E.K., Russ A.N., Das S., Fan Z., Muzikansky A., Garcia-Alloza M., Serrano-Pozo A. (2020). Increased mitochondrial calcium levels associated with neuronal death in a mouse model of Alzheimer’s disease. Nat. Commun..

[93] Takamura R., Mizuta K., Sekine Y., Islam T., Saito T., Sato M., Ohkura M., Nakai J., Ohshima T., Saido T.C. (2021). Modality-Specific Impairment of Hippocampal CA1 Neurons of Alzheimer’s Disease Model Mice. J. Neurosci..

[94] Busche M.A. (2018). In Vivo Two-Photon Calcium Imaging of Hippocampal Neurons in Alzheimer Mouse Models. Methods Mol. Biol..

[95] Sato M., Mizuta K., Islam T., Kawano M., Sekine Y., Takekawa T., Gomez-Dominguez D., Schmidt A., Wolf F., Kim K. (2020). Distinct Mechanisms of Over-Representation of Landmarks and Rewards in the Hippocampus. Cell Rep..

[96] Adams M.D., Celniker S.E., Holt R.A., Evans C.A., Gocayne J.D., Amanatides P.G., Scherer S.E., Li P.W., Hoskins R.A., Galle R.F. (2000). The genome sequence of Drosophila melanogaster. Science (80-.).

[97] Stocker H., Gallant P. (2008). Getting Started: An overview on raising and handling Drosophila. Methods Mol Biol..

[98] Hardie R.C., Minke B. (1992). The trp gene is essential for a light-activated Ca^2+^ channel in Drosophila photoreceptors. Neuron.

[99] Roos J., DiGregorio P.J., Yeromin A.V., Ohlsen K., Lioudyno M., Zhang S., Safrina O., Kozak J.A., Wagner S.L., Cahalan M.D. (2005). STIM1, an essential and conserved component of store-operated Ca^2+^ channel function. J. Cell Biol..

[100] Zhang K., Kaufman R.J. (2006). The unfolded protein response: A stress signaling pathway critical for health and disease. Neurology.

[101] Chorna T., Hasan G. (2012). The genetics of calcium signaling in Drosophila melanogaster. Biochim. Biophys. Acta Gen. Subj..

[102] DiAntonio A. (2006). Glutamate Receptors At The Drosophila Neuromuscular Junction. Int. Rev. Neurobiol..

[103] Yoshikawa S., Tanimura T., Miyawaki A., Nakamura M., Yuzaki M., Furuichi T., Mikoshiba K. (1992). Molecular cloning and characterization of the inositol 1,4,5-trisphosphate receptor in Drosophila melanogaster. J. Biol. Chem..

[104] Hasan G., Rosbash M. (1992). Drosophila homologs of two mammalian intracellular Ca^2+^-release channels: Identification and expression patterns of the inositol 1,4,5-triphosphate and the ryanodine receptor genes. Development.

[105] Takeshima H., Nishi M., Iwabe N., Miyata T., Hosoya T., Masai I., Hotta Y. (1994). Isolation and characterization of a gene for a ryanodine receptor/calcium release channel in *Drosophila melanogaster*. FEBS Lett..

[106] Bai J., Binari R., Ni J.-Q., Vijayakanthan M., Li H.-S., Perrimon N. (2008). RNA interference screening in *Drosophila* primary cells for genes involved in muscle assembly and maintenance. Development.

[107] Magyar A., Váradi A. (1990). Molecular cloning and chromosomal localization of a sarco/endoplasmic reticulum-type Ca^2+^-ATPase of drosophila melanogaster. Biochem. Biophys. Res. Commun..

[108] Southall T.D., Terhzaz S., Cabrero P., Chintapalli V.R., Evans J.M., Dow J.A., Davies S.A. (2006). Novel subcellular locations and functions for secretory pathway Ca^2+^/Mn2+-ATPases. Physiol. Genomics.

[109] Schwarz E.M., Benzer S. (1997). Calx, a Na-Ca exchanger gene of Drosophila melanogaster. Proc. Natl. Acad. Sci. USA.

[110] Brand A.H., Perrimon N. (1993). Targeted gene expression as a means of altering cell fates and generating dominant phenotypes. Development.

[111] Klueg K.M., Alvarado D., Muskavitch M.A.T., Duffy J.B. (2002). Creation of a GAL4/UAS-coupled inducible gene expression system for use indrosophila cultured cell lines. Genesis.

[112] Ito K., Shinomiya K., Ito M., Armstrong J.D., Boyan G., Hartenstein V., Harzsch S., Heisenberg M., Homberg U., Jenett A. (2014). A systematic nomenclature for the insect brain. Neuron.

[113] Raji J.I., Potter C.J. (2021). The number of neurons in Drosophila and mosquito brains. PLoS ONE.

[114] Scheffer L.K., Xu C.S., Januszewski M., Lu Z., Takemura S.Y., Hayworth K.J., Huang G.B., Shinomiya K., Maitin-Shepard J., Berg S. (2020). A connectome and analysis of the adult drosophila central brain. eLife.

[115] Cauchi R.J., Van Den Heuvel M. (2007). The fly as a model for neurodegenerative diseases: Is it worth the jump?. Neurodegener. Dis..

[116] Pandey U.B., Nichols C.D. (2011). Human disease models in drosophila melanogaster and the role of the fly in therapeutic drug discovery. Pharmacol. Rev..

[117] Simon A.F., Liang D.T., Krantz D.E. (2006). Differential decline in behavioral performance of Drosophila melanogaster with age. Mech. Ageing Dev..

[118] Markova O., Sénatore S., Chardès C., Lenne P.F. (2015). Calcium Spikes in Epithelium: Study on Drosophila early embryos. Sci. Rep..

[119] Vonhoff F., Keshishian H. (2017). *In Vivo* Calcium Signaling during Synaptic Refinement at the *Drosophila* Neuromuscular Junction. J. Neurosci..

[120] Karagyozov D., Mihovilovic Skanata M., Lesar A., Gershow M. (2018). Recording Neural Activity in Unrestrained Animals with Three-Dimensional Tracking Two-Photon Microscopy. Cell Rep..

[121] Marescotti M., Lagogiannis K., Webb B., Davies R.W., Armstrong J.D. (2018). Monitoring brain activity and behaviour in freely moving Drosophila larvae using bioluminescence. Sci. Rep..

[122] Lemon W.C., Pulver S.R., Höckendorf B., McDole K., Branson K., Freeman J., Keller P.J. (2015). Whole-central nervous system functional imaging in larval Drosophila. Nat. Commun..

[123] Pulver S.R., Bayley T.G., Taylor A.L., Berni J., Bate M., Hedwig B. (2015). Imaging fictive locomotor patterns in larval *Drosophila*. J. Neurophysiol..

[124] Dipt S., Riemensperger T., Fiala A. (2014). Optical calcium imaging using DNA-encoded fluorescence sensors in transgenic fruit flies, drosophila melanogaster. Methods Mol. Biol..

[125] Riemensperger T., Pech U., Dipt S., Fiala A. (2012). Optical calcium imaging in the nervous system of Drosophila melanogaster. Biochim. Biophys. Acta Gen. Subj..

[126] Ghaemi R., Rezai P., Nejad F.R., Selvaganapathy P.R. (2017). Characterization of microfluidic clamps for immobilizing and imaging of *Drosophila melanogaster* larva’s central nervous system. Biomicrofluidics.

[127] Tao X., Lin H.-H., Lam T., Rodriguez R., Wang J.W., Kubby J. (2017). Transcutical imaging with cellular and subcellular resolution. Biomed. Opt. Express.

[128] Huang C., Maxey J.R., Sinha S., Savall J., Gong Y., Schnitzer M.J. (2018). Long-term optical brain imaging in live adult fruit flies. Nat. Commun..

[129] Hampel S., Franconville R., Simpson J.H., Seeds A.M. (2015). A neural command circuit for grooming movement control. eLife.

[130] Shirangi T.R., Wong A.M., Truman J.W., Stern D.L. (2016). Doublesex Regulates the Connectivity of a Neural Circuit Controlling Drosophila Male Courtship Song. Dev. Cell.

[131] Hoopfer E.D., Jung Y., Inagaki H.K., Rubin G.M., Anderson D.J. (2015). P1 interneurons promote a persistent internal state that enhances inter-male aggression in Drosophila. eLife.

[132] Kaschula R., Salecker I. (2016). Neuronal Computations Made Visible with Subcellular Resolution. Cell.

[133] Yang P.C., Boras B.W., Jeng M.T., Docken S.S., Lewis T.J., McCulloch A.D., Harvey R.D., Clancy C.E. (2016). A Computational Modeling and Simulation Approach to Investigate Mechanisms of Subcellular cAMP Compartmentation. PLoS Comput. Biol..

[134] Chakraborty S., Hasan G. (2018). Store-Operated Ca^2+^ Entry in Drosophila Primary Neuronal Cultures. Methods Mol. Biol..

[135] Streit A.K., Fan Y.N., Masullo L., Baines R.A. (2016). Calcium Imaging of Neuronal Activity in Drosophila Can Identify Anticonvulsive Compounds. PLoS ONE.

[136] Vajente N., Norante R., Pizzo P., Pendin D. (2020). Calcium Imaging in Drosophila melanogaster. Calcium Signaling.

[137] Strube-Bloss M.F., Grabe V., Hansson B.S., Sachse S. (2017). Calcium imaging revealed no modulatory effect on odor-evoked responses of the Drosophila antennal lobe by two populations of inhibitory local interneurons. Sci. Rep..

[138] Silbering A.F., Bell R., Galizia C.G., Benton R. (2012). Calcium Imaging of Odor-evoked Responses in the Drosophila Antennal Lobe. J. Vis. Exp..

[139] Barnstedt O., Owald D., Felsenberg J., Brain R., Moszynski J.-P., Talbot C.B., Perrat P.N., Waddell S. (2016). Memory-Relevant Mushroom Body Output Synapses Are Cholinergic. Neuron.

[140] Heisenberg M. (2003). Mushroom body memoir: From maps to models. Nat. Rev. Neurosci..

[141] Menzel R. (2014). The insect mushroom body, an experience-dependent recoding device. J. Physiol..

[142] Honegger K.S., Campbell R.A.A., Turner G.C. (2011). Cellular-Resolution Population Imaging Reveals Robust Sparse Coding in the Drosophila Mushroom Body. J. Neurosci..

[143] Schnaitmann C., Haikala V., Abraham E., Oberhauser V., Thestrup T., Griesbeck O., Reiff D.F. (2018). Color Processing in the Early Visual System of Drosophila. Cell.

[144] Yorozu S., Wong A., Fischer B.J., Dankert H., Kernan M.J., Kamikouchi A., Ito K., Anderson D.J. (2009). Distinct sensory representations of wind and near-field sound in the Drosophila brain. Nature.

[145] Harris D.T., Kallman B.R., Mullaney B.C., Scott K. (2015). Representations of Taste Modality in the Drosophila Brain. Neuron.

[146] Patella P., Wilson R.I. (2018). Functional Maps of Mechanosensory Features in the Drosophila Brain. Curr. Biol..

[147] Mann K., Gallen C.L., Clandinin T.R. (2017). Whole-Brain Calcium Imaging Reveals an Intrinsic Functional Network in Drosophila. Curr. Biol..

[148] Grover D., Katsuki T., Greenspan R.J. (2016). Flyception: Imaging brain activity in freely walking fruit flies. Nat. Methods.

[149] Wangler M.F., Yamamoto S., Bellen H.J. (2015). Fruit Flies in Biomedical Research. Genetics.

[150] Michno K., Knight D., Campussano J.M., van de Hoef D., Boulianne G.L. (2009). Intracellular calcium deficits in Drosophila cholinergic neurons expressing wild type or FAD-mutant presenilin. PLoS ONE.

[151] Li M., Zhang W., Wang W., He Q., Yin M., Qin X., Zhang T., Wu T. (2018). Imidazole improves cognition and balances Alzheimer’s-like intracellular calcium homeostasis in transgenic Drosophila model. Neurourol. Urodyn..

[152] Higham J.P., Hidalgo S., Buhl E., Hodge J.J.L. (2019). Restoration of Olfactory Memory in Drosophila Overexpressing Human Alzheimer’s Disease Associated Tau by Manipulation of L-Type Ca^2+^ Channels. Front. Cell. Neurosci..

[153] Mahoney R., Ochoa Thomas E., Ramirez P., Miller H.E., Beckmann A., Zuniga G., Dobrowolski R., Frost B. (2020). Pathogenic Tau Causes a Toxic Depletion of Nuclear Calcium. Cell Rep..

[154] Garrido-Maraver J., Loh S.H.Y., Martins L.M. (2020). Forcing contacts between mitochondria and the endoplasmic reticulum extends lifespan in a Drosophila model of Alzheimer’s disease. Biol. Open.

[155] Nüsslein-Volhard C., Dahm R. (2002). Zebrafish: A Practical Approach.

[156] Kimmel C.B. (1993). Patterning the brain of the zebrafish embryo. Annu. Rev. Neurosci..

[157] Ahrens M.B., Li J.M., Orger M.B., Robson D.N., Schier A.F., Engert F., Portugues R. (2012). Brain-wide neuronal dynamics during motor adaptation in zebrafish. Nature.

[158] Panier T., Romano S.A., Olive R., Pietri T., Sumbre G., Candelier R., Debrégeas G. (2013). Fast functional imaging of multiple brain regions in intact zebrafish larvae using selective plane illumination microscopy. Front. Neural Circuits.

[159] Naumann E.A., Fitzgerald J.E., Dunn T.W., Rihel J., Sompolinsky H., Engert F. (2016). From Whole-Brain Data to Functional Circuit Models: The Zebrafish Optomotor Response. Cell.

[160] Portugues R., Feierstein C.E., Engert F., Orger M.B. (2014). Whole-Brain Activity Maps Reveal Stereotyped, Distributed Networks for Visuomotor Behavior. Neuron.

[161] Chen X., Mu Y., Hu Y., Kuan A.T., Nikitchenko M., Randlett O., Chen A.B., Gavornik J.P., Sompolinsky H., Engert F. (2018). Brain-wide Organization of Neuronal Activity and Convergent Sensorimotor Transformations in Larval Zebrafish. Neuron.

[162] Dal Maschio M., Donovan J.C., Helmbrecht T.O., Baier H. (2017). Linking Neurons to Network Function and Behavior by Two-Photon Holographic Optogenetics and Volumetric Imaging. Neuron.

[163] Fornetto C., Tiso N., Pavone F.S., Vanzi F. (2020). Colored visual stimuli evoke spectrally tuned neuronal responses across the central nervous system of zebrafish larvae. BMC Biol..

[164] Easter S.S., Nicola G.N. (1996). The development of vision in the zebrafish (Danio rerio). Dev. Biol..

[165] Brockerhoff S.E., Hurley J.B., Janssen-Bienhold U., Neuhauss S.C., Driever W., Dowling J.E. (1995). A behavioral screen for isolating zebrafish mutants with visual system defects. Proc. Natl. Acad. Sci. USA.

[166] Clark D.T. (1981). Visual Responses in the Developing Zebrafish (Brachydanio Rerio).

[167] Borla M.A., Palecek B., Budick S., O’Malley D.M. (2002). Prey capture by larval zebrafish: Evidence for fine axial motor control. Brain Behav. Evol..

[168] Van Tijn P., Kamphuis W., Marlatt M.W., Hol E.M., Lucassen P.J. (2011). Presenilin mouse and zebrafish models for dementia: Focus on neurogenesis. Prog. Neurobiol..

[169] Tomasiewicz H.G., Flaherty D.B., Soria J.P., Wood J.G. (2002). Transgenic zebrafish model of neurodegeneration. J. Neurosci. Res..

[170] Paquet D., Bhat R., Sydow A., Mandelkow E.M., Berg S., Hellberg S., Fälting J., Distel M., Köster R.W., Schmid B. (2009). A zebrafish model of tauopathy allows in vivo imaging of neuronal cell death and drug evaluation. J. Clin. Investig..

[171] Saleem S., Kannan R.R. (2018). Zebrafish: An emerging real-time model system to study Alzheimer’s disease and neurospecific drug discovery. Cell Death Discov..

[172] Sassen W.A., Köster R. (2015). A molecular toolbox for genetic manipulation of zebrafish. Adv. Genom. Genet..

[173] Hwang W.Y., Fu Y., Reyon D., Maeder M.L., Tsai S.Q., Sander J.D., Peterson R.T., Yeh J.R., Joung J.K. (2013). Efficient genome editing in zebrafish using a CRISPR-Cas system. Nat. Biotechnol..

[174] Turrini L., Fornetto C., Marchetto G., Mullenbroich M.C., Tiso N., Vettori A., Resta F., Masi A., Mannaioni G., Pavone F.S. (2017). Optical mapping of neuronal activity during seizures in zebrafish. Sci. Rep..

[175] Zon L.I., Peterson R.T. (2005). In vivo drug discovery in the zebrafish. Nat. Rev. Drug Discov..

[176] Stuart G.W., McMurray J.V., Westerfield M. (1988). Replication, integration and stable germ-line transmission of foreign sequences injected into early zebrafish embryos. Development.

[177] Kawakami K. (2007). Tol2: A versatile gene transfer vector in vertebrates. Genome Biol..

[178] Kettunen P. (2020). Calcium Imaging in the Zebrafish. Adv. Exp. Med. Biol..

[179] Brenner S. (1974). The genetics of Caenorhabditis elegans. Genetics.

[180] Corsi A.K., Wightman B., Chalfie M. (2015). A Transparent window into biology: A primer on Caenorhabditis elegans. WormBook.

[181] The *C. elegans* Sequencing Consortium (1998). Genome Sequence of the Nematode *C. elegans*: A Platform for Investigating Biology. Science (80-.).

[182] Kim W., Underwood R.S., Greenwald I., Shaye D.D. (2018). Ortholist 2: A new comparative genomic analysis of human and caenorhabditis elegans genes. Genetics.

[183] Lai C.H., Chou C.Y., Ch’ang L.Y., Liu C.S., Lin W.C. (2000). Identification of novel human genes evolutionarily conserved in Caenorhabditis elegans by comparative proteomics. Genome Res..

[184] Altun Z., Hall D. (2011). Nervous System General Description. WormAtlas.

[185] White J.G., Southgate E., Thomson J.N., Brenner S. (1986). The structure of the nervous system of the nematode Caenorhabditis elegans. Philos. Trans. R. Soc. Lond. B Biol. Sci..

[186] Cook S.J., Jarrell T.A., Brittin C.A., Wang Y., Bloniarz A.E., Yakovlev M.A., Nguyen K.C.Q., Tang L.T.H., Bayer E.A., Duerr J.S. (2019). Whole-animal connectomes of both Caenorhabditis elegans sexes. Nature.

[187] Li H., Avery L., Denk W., Hess G.P. (1997). Identification of chemical synapses in the pharynx of Caenorhabditis elegans. Proc. Natl. Acad. Sci. USA.

[188] Mclntire S.L., Jorgensen E., Kaplan J., Horvitz H.R. (1993). The GABAergic nervous system of Caenorhabditis elegans. Nature.

[189] Alfonso A., Grundahl K., McManus J.R., Rand J.B. (1994). Cloning and characterization of the choline acetyltransferase structural gene (cha-1) from *C. elegans*. J. Neurosci..

[190] Sulston J., Dew M., Brenner S. (1975). Dopaminergic neurons in the nematode Caenorhabditis elegans. J. Comp. Neurol..

[191] Horvitz H.R., Chalfie M., Trent C., Sulston J.E., Evans P.D. (1982). Serotonin and octopamine in the nematode Caenorhabditis elegans. Science (80-.).

[192] Bargmann C.I. (1998). Neurobiology of the Caenorhabditis elegans genome. Science.

[193] Xiao R., Xu X.Z.S. (2009). Function and regulation of TRP family channels in *C. elegans*. Pflug. Arch. Eur. J. Physiol..

[194] Baylis H.A., Furuichi T., Yoshikawa F., Mikoshiba K., Sattelle D.B. (1999). Inositol 1,4,5-trisphosphate receptors are strongly expressed in the nervous system, pharynx, intestine, gonad and excretory cell of Caenorhabditis elegans and are encoded by a single gene (itr-1). J. Mol. Biol..

[195] Sakube Y., Ando H., Kagawa H. (1993). Cloning and mapping of a ryanodine receptor homolog gene of Caenorhabditis elegans. Ann. N. Y. Acad. Sci..

[196] Maryon E.B., Coronado R., Anderson P. (1996). unc-68 encodes a ryanodine receptor involved in regulating *C. elegans* body-wall muscle contraction. J. Cell Biol..

[197] Hoon Cho J., Bandyopadhyay J., Lee J., Park C.S., Ahnn J. (2000). Two isoforms of sarco/endoplasmic reticulum calcium ATPase (SERCA) are essential in Caenorhabditis elegans. Gene.

[198] Kraev A., Kraev N., Carafoli E. (1999). Identification and functional expression of the plasma membrane calcium ATPase gene family from Caenorhabditis elegans. J. Biol. Chem..

[199] He C., O’Halloran D.M. (2014). Analysis of the Na+/Ca^2+^ exchanger gene family within the phylum Nematoda. PLoS ONE.

[200] Strange K., Yan X., Lorin-Nebel C., Xing J. (2007). Physiological roles of STIM1 and Orai1 homologs and CRAC channels in the genetic model organism Caenorhabditis elegans. Cell Calcium.

[201] Manning G. (2005). Genomic Overview of Protein Kinases. WormBook.

[202] Dong B., Valencia C.A., Liu R. (2007). Ca^2+^/calmodulin directly interacts with the pleckstrin homology domain of AKT1. J. Biol. Chem..

[203] Plowman G.D., Sudarsanam S., Bingham J., Whyte D., Hunter T. (1999). The protein kinases of Caenorhabditis elegans: A model for signal transduction in multicellular organisms. Proc. Natl. Acad. Sci. USA.

[204] Mello C., Kramer J., Stinchcomb D., Ambros V. (1992). Efficient gene transfer in *C. elegans*: Extrachromosomal maintenance and integration of transforming sequences. Trends Genet..

[205] Dickinson D.J., Goldstein B. (2016). CRISPR-based methods for caenorhabditis elegans genome engineering. Genetics.

[206] Fraser A.G., Kamath R.S., Zipperlen P., Martinez-Campos M., Sohrmann M., Ahringer J. (2000). Functional genomic analysis of *C. elegans* chromosome I by systematic RNA interference. Nature.

[207] García-Casas P., Alvarez-Illera P., Fonteriz R.I., Montero M., Alvarez J. (2021). Mechanism of the lifespan extension induced by submaximal SERCA inhibition in *C. elegans*. Mech. Ageing Dev..

[208] Patten S.A., Aggad D., Martinez J., Tremblay E., Petrillo J., Armstrong G.A.B., La Fontaine A., Maios C., Liao M., Ciura S. (2017). Neuroleptics as therapeutic compounds stabilizing neuromuscular transmission in amyotrophic lateral sclerosis. JCI Insight.

[209] Ikenaka K., Tsukada Y., Giles A.C., Arai T., Nakadera Y., Nakano S., Kawai K., Mochizuki H., Katsuno M., Sobue G. (2019). A behavior-based drug screening system using a Caenorhabditis elegans model of motor neuron disease. Sci. Rep..

[210] Caldwell K.A., Willicott C.W., Caldwell G.A. (2020). Modeling neurodegeneration in Caenorhabditis elegans. DMM Dis. Model. Mech..

[211] Liang J.J.H., McKinnon I.A., Rankin C.H. (2020). The contribution of *C. elegans* neurogenetics to understanding neurodegenerative diseases. J. Neurogenet..

[212] Chung S.H., Sun L., Gabel C.V. (2013). In vivo neuronal calcium imaging in *C. elegans*. J. Vis. Exp..

[213] Martin A.A., Alford S., Richmond J.E. (2019). In vivo calcium imaging in *C. elegans* body wall muscles. J. Vis. Exp..

[214] Shigyou K., Maeoka H., Igarashi R., Sugi T. (2021). Calcium Imaging in Freely Behaving *Caenorhabditis elegans* with Well-Controlled, Nonlocalized Vibration. J. Vis. Exp..

[215] Shipley F.B., Clark C.M., Alkema M.J., Leifer A.M. (2014). Simultaneous optogenetic manipulation and calcium imaging in freely moving *C. elegans*. Front. Neural Circuits.

[216] Alvarez-Illera P., García-Casas P., Arias-del-Val J., Fonteriz R.I., Alvarez J., Montero M. (2017). Pharynx mitochondrial [Ca^2+^] dynamics in live *C. elegans* worms during aging. Oncotarget.

[217] Garcia-Casas P., Alvarez-Illera P., Gomez-Orte E., Cabello J., Fonteriz R., Montero M., Alvarez J. (2021). The Mitochondrial Na+/Ca^2+^ Exchanger inhibitor CGP37157 preserves muscle structure and function to increase lifespan and healthspan in Caenorhabditis elegans. Front. Pharmacol..

[218] Sarasija S., Laboy J.T., Ashkavand Z., Bonner J., Tang Y., Norman K.R. (2018). Presenilin mutations deregulate mitochondrial Ca^2+^ homeostasis and metabolic activity causing neurodegeneration in Caenorhabditis elegans. eLife.

[219] Nguyen J.P., Shipley F.B., Linder A.N., Plummer G.S., Liu M., Setru S.U., Shaevitz J.W., Leifer A.M. (2016). Whole-brain calcium imaging with cellular resolution in freely behaving *Caenorhabditis elegans*. Proc. Natl. Acad. Sci. USA.

[220] Venkatachalam V., Ji N., Wang X., Clark C., Mitchell J.K., Klein M., Tabone C.J., Florman J., Ji H., Greenwood J. (2016). Pan-neuronal imaging in roaming *Caenorhabditis elegans*. Proc. Natl. Acad. Sci. USA.

[221] Alvarez J., Alvarez-Illera P., García-Casas P., Fonteriz R.I., Montero M. (2020). The Role of Ca^2+^ Signaling in Aging and Neurodegeneration: Insights from Caenorhabditis elegans Models. Cells.

[222] Alvarez-Illera P., Sanchez-Blanco A., Lopez-Burillo S., Fonteriz R.I., Alvarez J., Montero M. (2016). Long-term monitoring of Ca^2+^ dynamics in *C. elegans* pharynx: An *in vivo* energy balance sensor. Oncotarget.

[223] Link C.D. (2006). *C. elegans* models of age-associated neurodegenerative diseases: Lessons from transgenic worm models of Alzheimer’s disease. Exp. Gerontol..

[224] Newman M., Ebrahimie E., Lardelli M. (2014). Using the zebrafish model for Alzheimerâ€^TM^s disease research. Front. Genet..

[225] Chang Y., Kim J., Park H., Choi H., Kim J. (2020). Modelling neurodegenerative diseases with 3D brain organoids. Biol. Rev..

[226] Wray S. (2021). Modelling neurodegenerative disease using brain organoids. Semin. Cell Dev. Biol..

[227] Grenier K., Kao J., Diamandis P. (2020). Three-dimensional modeling of human neurodegeneration: Brain organoids coming of age. Mol. Psychiatry.

